# Allogeneic Bone Marrow Transplant from MRL/MpJ Super-Healer Mice Does Not Improve Articular Cartilage Repair in the C57Bl/6 Strain

**DOI:** 10.1371/journal.pone.0131661

**Published:** 2015-06-29

**Authors:** Catherine A. Leonard, Woo-Yong Lee, Pankaj Tailor, Paul T. Salo, Paul Kubes, Roman J. Krawetz

**Affiliations:** 1 McCaig Institute for Bone and Joint Health, Department of Surgery, University of Calgary, Calgary, Alberta, Canada; 2 Calvin, Phoebe, and Joan Snyder Institute for Chronic Disease, Department of Physiology and Pharmacology, University of Calgary, Calgary, Alberta, Canada; INSERM-Université Paris-Sud, FRANCE

## Abstract

**Background:**

Articular cartilage has been the focus of multiple strategies to improve its regenerative/ repair capacity. The Murphy Roths Large (MRL/MpJ) “super-healer” mouse demonstrates an unusual enhanced regenerative capacity in many tissues and provides an opportunity to further study endogenous cartilage repair. The objective of this study was to test whether the super-healer phenotype could be transferred from MRL/MpJ to non-healer C57Bl/6 mice by allogeneic bone marrow transplant.

**Methodology:**

The healing of 2mm ear punches and full thickness cartilage defects was measured 4 and 8 weeks after injury in control C57Bl/6 and MRL/MpJ “super-healer” mice, and in radiation chimeras reconstituted with bone marrow from the other mouse strain. Healing was assessed using ear hole diameter measurement, a 14 point histological scoring scale for the cartilage defect and an adapted version of the Osteoarthritis Research Society International scale for assessment of osteoarthritis in mouse knee joints.

**Principal Findings:**

Normal and chimeric MRL mice showed significantly better healing of articular cartilage and ear wounds along with less severe signs of osteoarthritis after cartilage injury than the control strain. Contrary to our hypothesis, however, bone marrow transplant from MRL mice did not confer improved healing on the C57Bl/6 chimeras, either in regards to ear wound healing or cartilage repair.

**Conclusion and Significance:**

The elusive cellular basis for the MRL regenerative phenotype still requires additional study and may possibly be dependent on additional cell types external to the bone marrow.

## Introduction

The Murphy Roths Large (MRL/MpJ “MRL”) mouse was initially bred for its large size, but also demonstrates an unusual enhanced tissue regenerative capacity and has become known as the “super-healer” mouse (for recent reviews, see references [1, 2]). Enhanced healing was first reported for closing of the ear hole punches that are used as a standard identification system for mouse colonies: Clark et al [3] found that 2mm diameter ear punches in MRL mice regenerated within 4 weeks without scarring or fibrosis, in a manner similar to embryonic epidermal healing. Since then, it has been found that multiple MRL tissues have enhanced healing ability, including heart [4], cornea [5] and articular cartilage [6,7]. MRL mice are also resistant to developing post-traumatic osteoarthritis (OA) following intra-articular fracture [8,9].

Not all studies have agreed upon the regenerative capacity of MRL tissues, even in similar injury models. For example, Colwell et al. [10] studied the healing of full-thickness skin wounds on the dorsum of 7-week-old MRL/MpJ mice and found that (in contrast to wound healing in the ear) the dorsal wounds healed with scar formation and dermal collagen deposition, similarly to control C57Bl/6 mice. In contrast, Tolba et al. [11] showed that MRL skin graft recipients had increased neovascularization, a blunted inflammatory response and improved stem cell recruitment to the grafted area. There are other conflicting reports of regeneration versus scarring in the same tissue, most notably the myocardium [4, 12].

At the core of most MRL studies, there is a consensus that this mouse strain does exhibit an enhanced repair capacity and some groups have attempted to isolate the cells and/or genes that are responsible for this ‘healer’ phenotype [13]. Bone marrow stromal cells are the progenitors of skeletal tissue components such as bone, muscle, skin and cartilage: green fluorescent protein bone marrow chimeric mice have been used to show the involvement of bone-marrow derived cells in tissue repair [14, 15, 16]. A logical next step after discovery of the super-healer mouse was therefore to test whether the healing phenotype could be transferred by allogeneic bone marrow transplantation from MRL to non-healer mice. The results of these studies showed that the ear-hole closing phenotype appears to be robustly recipient-dependent [4, 17]; however, some donor MRL phenotype transfer has been observed with fetal liver cell transplant and cryoinjury to the heart [18].

Our hypothesis that reconstitution of lethally irradiated B6 mice with super-healer MRL bone marrow would confer the healing phenotype and enhance articular cartilage regeneration in the chimeras was based on previous studies showing that:

1. Healing of small full-thickness cartilage defects is initiated by the proliferation and differentiation of marrow-derived cells rather than by repopulation with chondrocytes from adjacent residual articular cartilage [19, 20].
2. Bone marrow mesenchymal cells contribute to wound healing in skin, muscle and bone: transplants of GFP-positive bone marrow result in fluorescent donor-derived cells at the wound sites in B6 recipients [15, 16].
3. MRL/lpr chimeras that received bone marrow transplants from B6 mice showed donor-derived Type a synoviocytes (macrophages) in their knee joints [21].
4. Cartilage healing can be enhanced by intra-articular injection of mesenchymal stem cells (for review see reference [22; 23]).
5. Cell transfers from MRL mice to non-healer strains can result in an increased repair capacity in certain tissues [18].
6. Blood-borne factors in the systemic milieu of mice can influence cellular generation and function when transplanted into mice of different ages [24].

In non-healer mice, articular cartilage has a very poor regenerative capacity. Depending on their size, full thickness cartilage injuries generally show partial filling of the wound site with fibrocartilaginous tissue that arises from rapid migration of marrow-derived undifferentiated cells into the defect [19]. Tissue regenerative capacity in mice has a strong heritable genetic component and there is a clear correlation between ear healing and articular cartilage regeneration in multiple strains [25]. The current study tested whether the enhanced cartilage repair phenotype observed in MRL mice can be transferred to C57Bl/6 mouse cartilage by allogeneic bone marrow transplant from MRLs.

## Methods

### Ethics Statement

This study was carried out in accordance with the recommendations in the Canadian Council on Animal Care Guidelines. Animal protocols and surgical procedures were approved by the University of Calgary Animal Care Committee (protocol AC12-0031). All surgery was performed under isoflurane anaesthesia and mice were given post-surgical buprenorphine (Abbott Animal Health, 0.05mg/kg i.p.) for analgesia.

### Animals

Groups of 6 week old male C57Bl/6 “(B6”) and MRL/MpJ (“MRL”) mice were purchased from Jackson Laboratories (Bar Harbor, Maine) and housed 5 per cage on a 12h light/dark cycle. Groups of mice were used as shown in Table 1, plus 3 additional mice of each strain as bone marrow donors. Full thickness cartilage defects (FTCD) and ear punches were created unilaterally and mice were sacrificed at 4 or 8 weeks after cartilage injury.

**Table 1:** Animal Groups and Protocol.

Group	Experimental Protocol	Number of animals
1 B6 Control	FTCD	15
2 B6 Chimera	Bone marrow transplant from MRL mice plus FTCD	16
3 MRL Control	FTCD	16
4 MRL Chimera	Bone marrow transplant from B6 mice plus FTCD	23

### Creation of Bone Marrow Chimeras and FACS Analysis

B6 and MRL bone marrow chimeras were generated according to our previously described standard protocol [26]. Lethally irradiated recipients were prepared by exposure to 2 doses of 500 rad (Gammacell 40 ^137^Cs γ-irradiation source), with an interval of 3 hours between the first and second irradiations. This protocol has previously been shown to destroy 99% of existing bone marrow cells and untreated animals die within 30 days. Donor bone marrow was isolated from the tibia, femur and pelvic bones of 8-week-old mice and resuspended in sterile Hank’s balanced salt solution (Sigma) at a concentration of 8x10^6^ cells/mL. Each irradiated recipient was given 0.2 mL of cell suspension from the other mouse strain via tail vein injection. Following transplant, mice were given 2% neomycin (Sigma) in their drinking water for 2 weeks.

**Fig 1:**
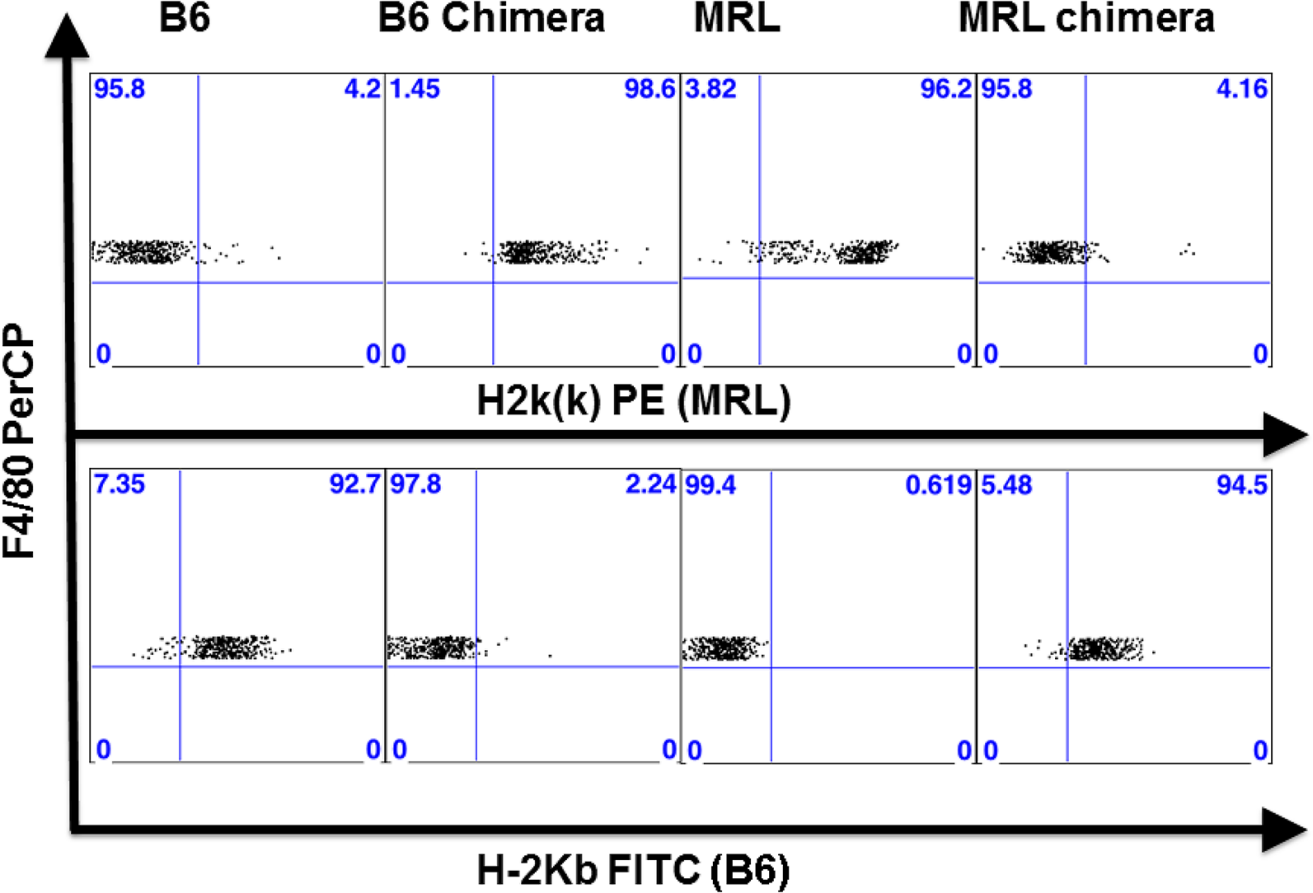
Confirmation of Chimerism in Mice. Representative FACS plots from wild-type (B6, MRL) and chimeric mice (B6 Chimera, MRL Chimera).Chimeras were identified by MHC H2Kk (upper panel) or MHC H2kb (lower panel) reactivity on F4/80 positive cells from the spleen. In the chimeric mice, ∼90% of the F4/80 cells were positive for H2K markers originating from the donor strain.

Chimerism of bone-marrow reconstituted recipient mice was confirmed when the animals were killed after 14 weeks (6 weeks engraftment plus 8 weeks cartilage healing) by flow cytometric analysis of spleen cells for strain-specific Class 1 MHC H2K markers (antibodies from BD Bioscience, used at 1:50). Cells were labelled with anti-mouse F4/80 PerCP- Cy 5.5 (eBioscience, used at 1:50), anti-mouse H2kk PE and anti-mouse H2Kb FITC. All chimeric mice showed >90% of F4/80 cells derived from donor bone marrow as determined by H2Kb (B6) or H2kk (MRL) staining (Fig 1).

### Cartilage Injury and Ear Wound Models

Standardized cartilage injuries were created with the mouse under isoflurane (Baxter) anesthesia (1.5% v/v O_2_). A small medial para-patellar arthrotomy was performed under a dissection microscope (Leica). A full-thickness cartilage defect (FTCD) was created using a custom made device in which an epoxy bead was attached 200μm from the tip of a 26G needle to control the depth of the injury. The tip of the needle was used to gently displace the patella laterally, placed in the femoral groove anterior to the intercondylar notch and rotated to make a circular wound approximately 0.6mm in diameter that penetrated into the underlying subchondral bone. The patellar dislocation was then reduced and the skin closed with a wound clip.

### Ear Wound Generation and Quantification of Healing

While mice were anaesthetized for cartilage injury, 2mm diameter circular wounds were punched in the central portion of the right ear pinna using a metal thumb punch. Ear wounds in all mice were photographed along with a size marker at 0, 1, 2, 3, 4 and 8 weeks post-injury and the mean diameter from 3 repeat measures was determined from enlarged photos. Any wounds that healed in an irregular shape or became notched were not included in the data analysis.

### Cartilage Defect Healing and Osteoarthritis Scoring

Four weeks after FTCD, entire intact knee joints were dissected and fixed in 4% neutral buffered formalin (Sigma) for 24h. Samples were decalcified using Accumet “RDO” decalcifying solution (Sigma) for 2h, returned to neutral buffered formalin for a further 3 days and then processed for paraffin sectioning. Eight μm sagittal serial sections across the entire joint were cut on a microtome (Leica) and mounted on Superfrost Plus slides (Fisher Scientific). Every 5^th^ section across the joint was stained with safranin-O, fast green and haematoxylin (all Electron Microscopy Sciences) to visualize proteoglycans, collagen and cell nuclei respectively.

Cartilage repair in all mouse groups was evaluated after 4 weeks by scoring the full-thickness defect for cell morphology (0–4), matrix staining (0–3), surface regularity (0–3), thickness of cartilage (0–2) and integration with native cartilage (0–2) (see reference 6). On this scale, a newly created FTCD has an overall score of 0 and the (maximal) overall score for uninjured native cartilage is 14. The cell morphology score (0–4) is an indicator of the type of tissue that has filled the defect: a score of 4 indicates complete regeneration of smooth-surfaced hyaline cartilage, while lower scores are given for defects filled with fibrocartilaginous or non-cartilage tissue. The matrix staining score (0–3) measures the amount of proteoglycan present in the cartilage defect: a score of 3 indicates dense continuous staining throughout the thickness of the cartilage as in uninjured joints.

In all four animal groups, the entire joint was graded for signs of OA using a modification of the Osteoarthritis Research Society International Guidelines for mouse knee joints [27]. The original semi-quantitative grading scale uses frontal sections and scores all four cartilage areas on the same section. Since knees in the current study were sectioned sagittally to allow for prior cartilage injury grading, the medial femoral condyle, medial tibial plateau, lateral femoral condyle and lateral tibial plateau were assessed by scoring every 5^th^ section across the entire joint. The grading scale was identical to that in the OARSI guidelines and ranged from 0–6 based upon the following structural changes to the cartilage in each area of interest:

0 Normal
0.5 Loss of Safranin-O without structural changes
1 Small fibrillations without loss of cartilage
2 Vertical clefts in the superficial layer only and some loss of surface lamina;
3 Vertical clefts/erosion to the calcified cartilage extending to <25% of the articular surface;
4 Vertical clefts/erosion to the calcified cartilage extending to 25–50% of the articular surface;
5 Vertical clefts/erosion to the calcified cartilage extending to 50–75% of the articular surface;
6 Vertical clefts/erosion to the calcified cartilage extending >75% of the articular surface.

Standardized specimen histological sections demonstrating the use of this grading scale have been previously published [27].

### Immunohistochemistry

Sagittal sections of whole knee joints in the area of the defect were labeled using a standard TRIS buffer immunohistochemistry protocol to identify the MRL-specific marker Class 1 MHC H2Kk. Briefly, sections were deparaffinized, then washed and permeabilized in TRIS buffer / 0.1% Triton-X 100 for 45min (all Sigma). Antigen retrieval (Proteinase K, 30min at 37° C, Life Technologies), blocking (10% normal goat serum and 1% bovine serum albumin, both from Sigma) and endogenous biotin blocking steps (15min egg white, 10% w/v in water and 15 min skim milk blocking, all from Sigma) were performed and the sections were incubated overnight at 4°C with a biotin-conjugated primary antibody to Class 1 MHC H2Kk (1:200; Abcam). The primary antibody was visualized using streptavidin-HRP (1:500 for 2h at room temperature, Sigma) followed by exposure to DAB (Sigma), 2.5-3min, or by streptavidin-PE (1:500 for 2h at room temperature, Bio-Rad).

### Statistics

GraphPad Prism software (Version 4.3) was used to summarize the variables (means and standard deviation) and perform standard descriptive statistics. p ≤ 0.05 was considered to be significant. Statistical analysis was performed by ANOVA followed by Bonferroni’s post hoc testing. Statistical methods were reviewed by a biostatistician at the University of Calgary.

## Results

### Chimeras

**Fig 2:**
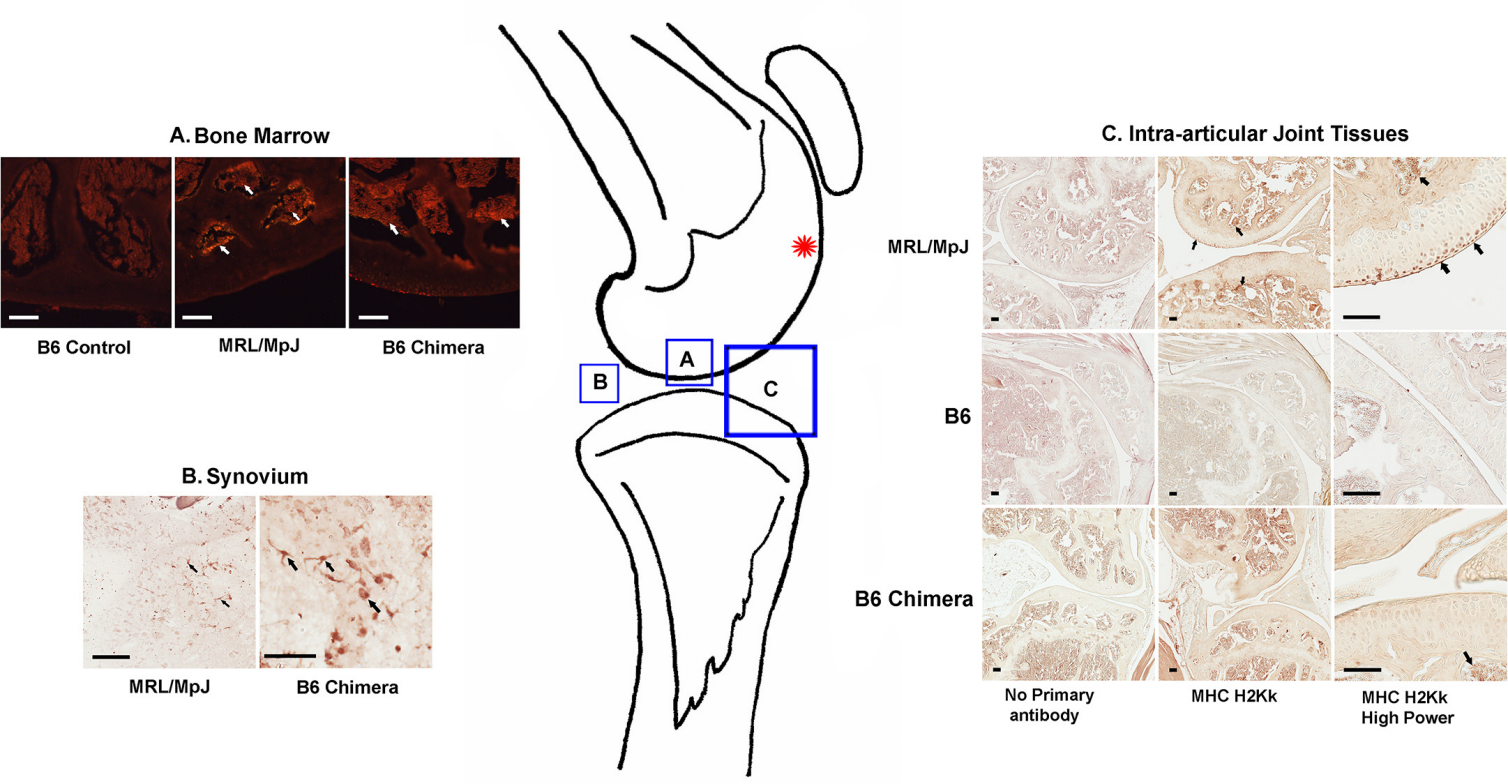
Immunostaining of MRL-specific markers in B6 chimeric mice. Knee joint tissues of MRL control, B6 control and B6 chimeric mice are immunopositive for Class 1 MHC H2Kk (arrows indicate typical immunopositive cells). Location of the cartilage injury in the femoral groove is indicated by the red asterisk. Scale bars all = 100μm. A. Bone marrow: engraftment of MRL/MpJ bone marrow into B6 chimeric mice shown by the presence of MHC H2Kk-immunopositive cells (arrows). B: MRL-derived H2Kk-positive cells are seen in the synovium of B6 chimeric mice (arrows); C: MRL-derived cells are present in bone marrow of B6 chimeras but not in cartilage or other intra-articular tissues (bottom right panel).

Fourteen lethally irradiated B6 chimeras and 19 MRL chimeras survived the 6 week engraftment period. In addition to FACS analysis presented in Fig 1, chimerism of the surviving B6 recipients was confirmed by positive immunohistochemical staining for MRL-origin marker cells in their bone marrow and knee joint synovium (Fig 2; [28]).

### Ear wound healing

**Fig 3:**
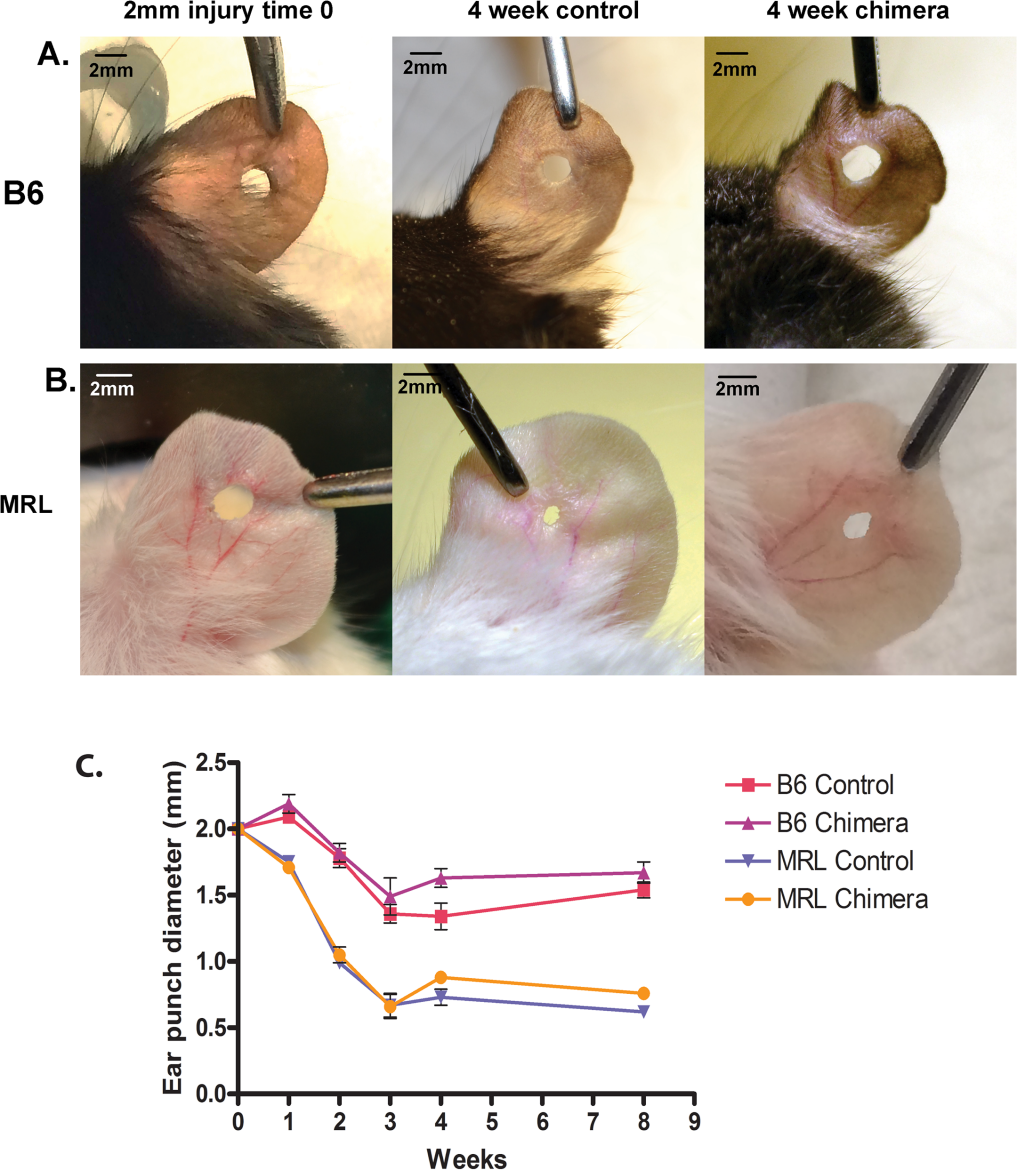
Ear wound healing. Representative images of ear wound closing in B6 (Row A) and MRL (Row B) control and chimeric mice at 0 and 4 weeks after injury. Scale bar = 1mm. Graph C summarizes the mean diameter of initial 2mm ear punch holes over time. Error bar in the B6 control group is not visible due to its small size.

Eleven out of 70 total ear punches healed in an irregular fashion and were not included in the data analysis. In the control mouse groups, the initial 2mm ear punches were reduced in size after 4 weeks of healing to mean diameters as follows: B6 controls, 1.34 ± 0.08mm; B6 chimeras, 1.63 ± 0.0mm; MRL controls, 0.73 ± 0.06mm; MRL chimeras, 0.88 ± 0.04mm (Fig 3). There was no significant further reduction in ear punch diameter in any of the groups between 4 and 8 weeks (Fig 3C). The MRL mice showed significantly better healing than the B6 mice (as expected, p<0.01). Reconstitution of lethally irradiated B6 mice with MRL bone marrow did not improve the closing of ear punch wounds in the chimeras; in fact, ear wound closing was slightly worse than in B6 controls at 4 weeks (Fig 3 Row A and Graph C).

### Cartilage injury and repair

**Fig 4:**
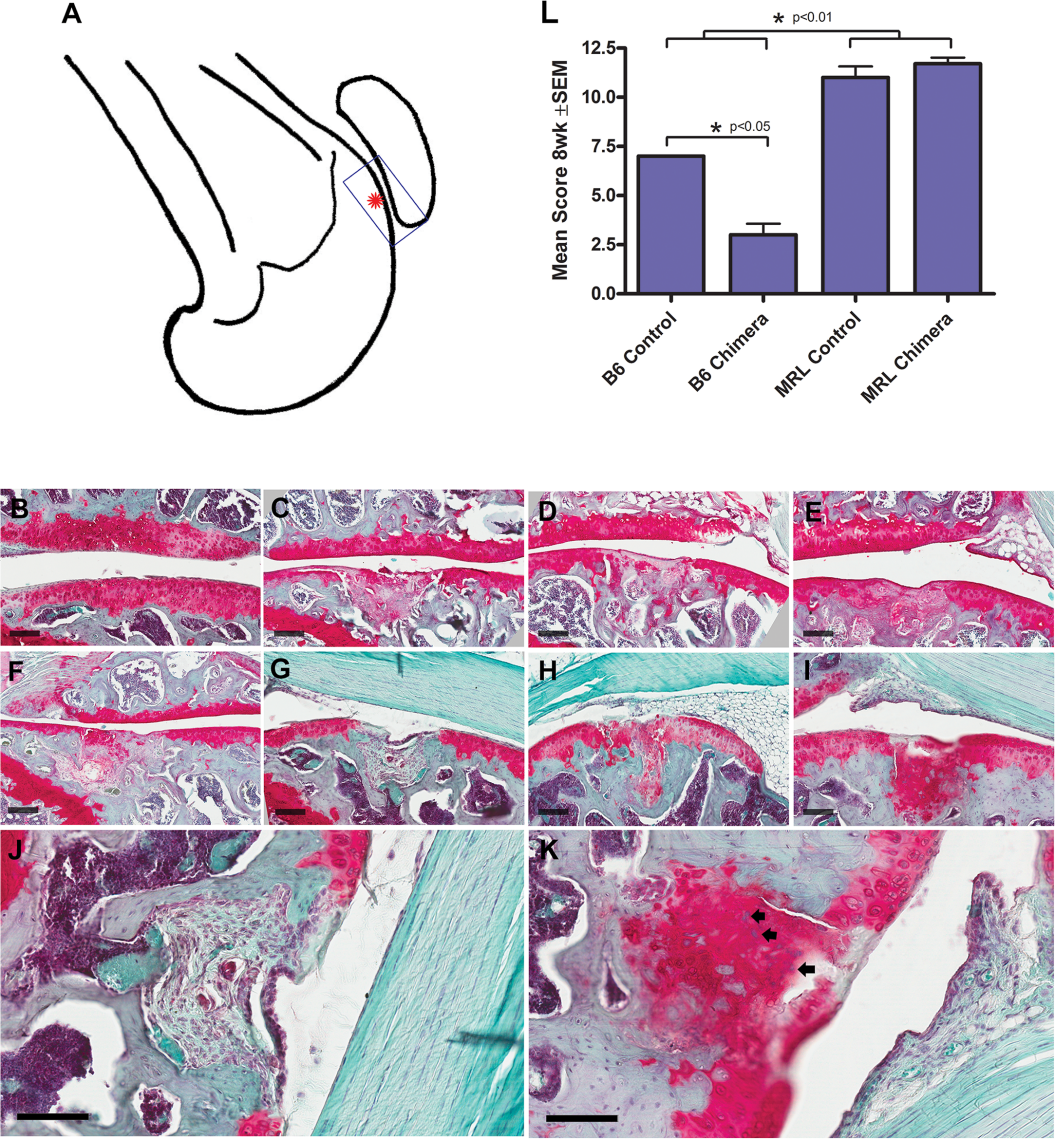
Cartilage defect healing in C57Bl/6 and MRL/MpJ mice. A: Schematic of mouse femur showing location of the cartilage defect (asterisk) and the area of the joint enlarged in panels B-K. B-I show representative healing cartilage defects stained with haematoxylin, fast green and Safranin-O. Scale bars = 100μm. B: Uninjured cartilage. C, D, E: 4 week healing cartilage from B6 control, B6 chimera and MRL control mice respectively. F, G, H, I: 8 week healing cartilage defects from B6 control, B6 chimera, MRL control and MRL chimeric mice, respectively. J and K: High power images of 8 week cartilage defects from panels G (B6 chimera) and I (MRL chimera) respectively. Arrows in K indicate MRL cells surrounded by newly formed proteoglycan matrix that are not seen in B6 mice (J). Graph L shows the average histological scores for the 4 animal groups (asterisk indicates statistically significant difference in all MRL vs all B6 mice, p<0.01).

We first confirmed the previously published result of Fitzgerald et al. [6] that MRL/MpJ mice can repair their articular cartilage significantly better than B6 mice (Fig 4). At 4 weeks after injury, the mean histological score was 6.7 ± 0.2 in MRLs vs. 4.9 ± 0.2 in B6s, p<0.01. By 8 weeks, the mean score in MRLs had increased to 11.0 ± 0.57 vs 7.0 ± 0.03 in B6s (see graph Fig 4L). All FTCDs showed filling of the wound site with non-cartilage or fibrocartilaginous cells and reduced regularity of the surface. However, MRL mice showed increased matrix staining (Fig 4J and 4K) along with significant hypertrophy of the cartilage in the region immediately surrounding the injury site in 6 of the 10 animals. After 8 weeks of cartilage healing, MRL chimeras showed no significant difference in healing score from control MRLs, and B6 chimeras surprisingly showed significantly worse healing than B6 controls (Fig 4L).

**Fig 5:**
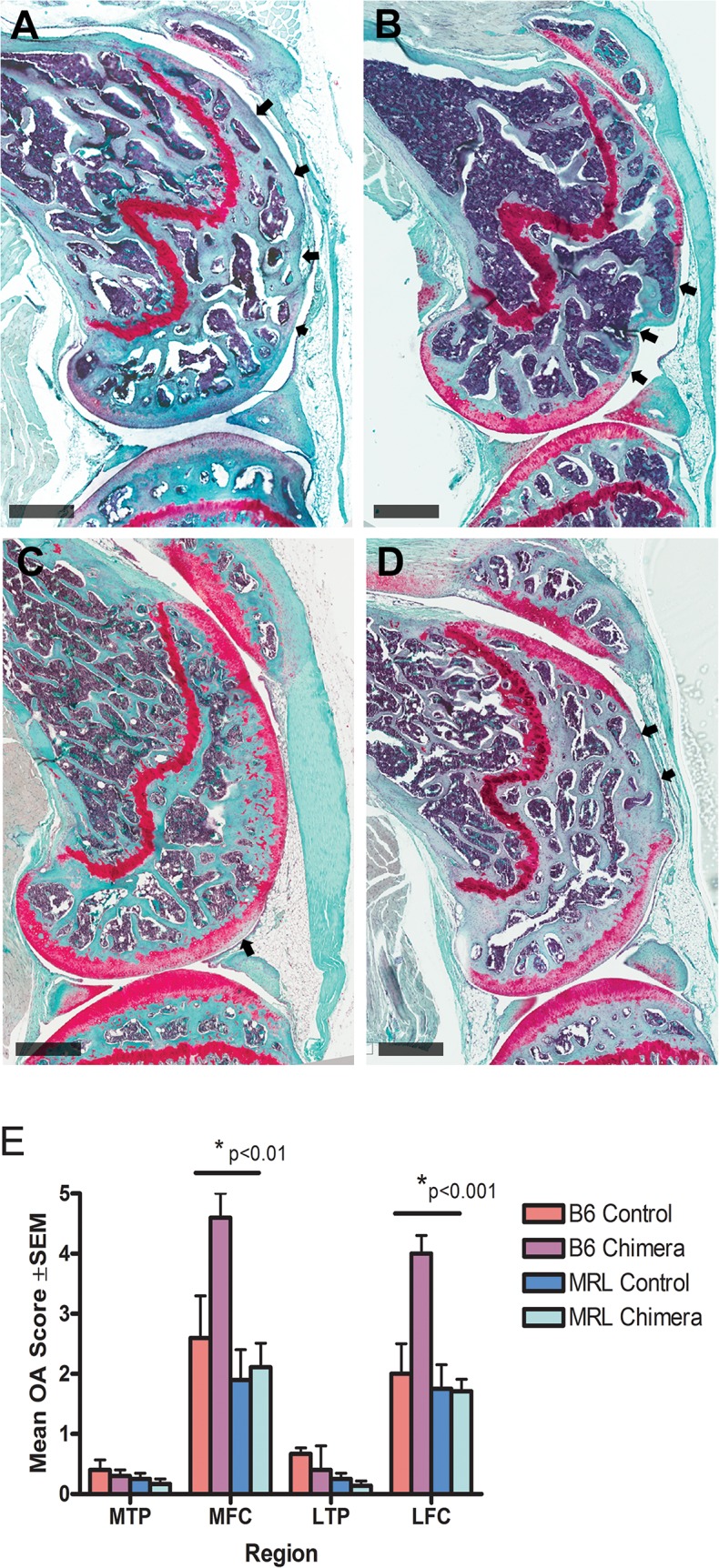
Progression of osteoarthritis in injured mouse knee joints. A-D: Sagittal sections of mouse femurs stained with haematoxylin, fast green and Safranin-O 8 weeks after injury to the femoral cartilage. Scale bars = 500μm. A: B6 control. B: B6 chimera. C: MRL Control. D: MRL chimera. Arrows in A, B and D indicate regions of complete cartilage loss. Arrow in C indicates a region of reduced proteoglycan staining. Graph E shows whole joint mean OA scores 8 weeks after full thickness cartilage defect injury in B6 and MRL controls and chimeras. Max OA score = 6. Scoring was performed on sections lateral and medial to the defect area and away from ligament insertions. Medial tibial plateau (MTP), medial femoral condyle (MFC), lateral tibial plateau (LTP) and lateral femoral condyle (LFC). The lateral and medial femoral condyles in B6 chimeras showed significantly higher OA scores than any other group (p<0.01 for MFC and p<0.001 for LFC).

To assess whole joint degradative effects of surgery and the cartilage defect, we used a standard OA grading scale for mouse [27]. The results from 8 weeks after injury are shown in Fig 5. All the mice had very low mean OA scores for the lateral and medial tibial plateaus, where the cartilage appeared normal and showed only occasional minor loss of proteoglycan staining. The femoral condyles in all 4 groups showed degradative changes at 4 and 8 weeks, but particularly in the B6 chimeras, where we observed significant loss of cartilage in both lateral and medial compartments (mean 8 week OA scores were 4.6 ± 0.4 for MFC and 4.0 ± 0.3 for LFC). At 4 weeks after injury the MRL healing phenotype conferred some protective effect in the femoral condyles compared to B6 controls (data not shown), but by 8 weeks the OA scores in MRL and B6 control animals were not significantly different.

## Discussion

Since OA and degenerative joint conditions are a significant drain on North American health care resources [29], articular cartilage has been the focus of multiple strategies to improve its regenerative capacity, particularly through intra-articular transplant of stem and progenitor cells [22]. The super-healer MRL mouse provides an opportunity to further elucidate the mechanisms by which endogenous cells contribute to wound healing. To our knowledge, articular cartilage healing in C57Bl/6 –MRL/MpJ bone marrow chimeric mice has not been measured.

Previous studies have attempted to transfer the super-healer phenotype from MRL to B6 mice with varying degrees of success and the aim of the current study was to examine articular cartilage healing in the same mouse model used for studies of regeneration in heart and other tissues [4, 5]. The initial intent was not to distinguish haematopoietic versus mesenchymal stem cell contribution to cartilage healing (since both populations are transferred with our bone marrow transplant protocol) but to quantify cartilage repair in the chimeras and determine if further analysis of contributing cell populations was warranted.

We first confirmed that the super-healer mouse shows better intrinsic regeneration of both ear punch wounds and articular cartilage (Figs 3 and 4) as originally noted by Fitzgerald et al [6]. MRL cartilage showed a significant improvement in histological score over B6 mice at 8 weeks after a full thickness cartilage defect injury. In several of the MRL mice we also observed large numbers of hypertrophic, proteoglycan-positive cells in the defect (Fig 4J) which indicate either enhanced division of locally-derived cells, or greater recruitment of progenitor cells to the injury site. In a recent review of the MRL mouse strain, Heydemann [1] suggested alterations in cell cycle and proliferation, stem cells, immune response, remodelling or metabolism as potential healing mechanisms in super healers. Since MRLs have reduced expression of the cell cycle control gene p21 [30], and evidence has been presented suggesting that cartilage defect repair is initiated by migration of undifferentiated mesenchymal cells into the defect [31], we propose that cell cycle changes or differences in stem cell biology are most likely responsible for the super-healer phenotype; however, more complex experimentation including transgenic models will be required to prove this hypothesis.

It has previously been shown that MRLs are protected from post-traumatic arthritis after closed intra-articular fracture [8, 9]. The authors saw no difference in bone density, subchondral bone thickness or histologic grading of cartilage degeneration between fractured and contralateral control limbs in MRL mice. Although our injury model is different than other published OA models, MRL mice in the current study showed an initial (4 week) improvement in OA score for femoral cartilage compared to B6 controls, indicating that the super-healer mice are better able to maintain the structural integrity of their cartilage for some time following surgical opening of the joint capsule. By 8 weeks after injury, the difference was no longer apparent.

Contrary to our hypothesis, bone marrow transplant from MRL mice did not confer an improved healing phenotype on the B6 chimeras, either in ear wound healing or cartilage repair. We observed MHC H2Kk positive, MRL-derived cells in the synovial niche of the B6 chimeric mice and believe that these are derived from the transplanted hematopoietic stem cells (HSC). Based on cell morphology and the previous study by Kitegawa et al [21], these MRL-derived synovial cells appear to be macrophages and/or dendritic cells. Based upon lack of MHC H2Kk labelling in the injured articular cartilage of the B6 chimeras, we concluded that MRL MSCs are not contributing to cartilage regeneration in this model at 4 or 8 weeks post-injury. Other reports using injury models with better native healing capacity than cartilage (such as skin and bone) clearly indicate incorporated donor MSCs in skin and muscle injury sites [14, 15, 16]. Improved healing of joint tissues by localized injection of purified MSCs has also been reported [22, 23]; however our results show that systemic delivery of exogenous donor cells produces a different outcome than injection directly into the joint. Barry and Murphy [22] have proposed that paracrine signalling by MSCs might be more important than differentiation in stimulating repair, and the relative contribution of these two processes may differ in the isolated environment of the joint.

A previously unreported finding of the current study was that the ear punch wounds in B6 chimeras actually healed less well than B6 control mice, while there was no significant difference in ear punch diameter between MRL controls and chimeras. Irradiation causes animal morbidity through a massive inflammatory response elicited by tissue damage, and bone marrow recipient mice must therefore survive a significant systemic insult. Interestingly, the “super-healer” MRL mice were better able to cope with this insult, demonstrating a lower death rate during the engraftment period than the B6 mice (4 MRL vs. 9 B6), and appearing in generally more robust health. The reduced healing rate in the ears of B6 chimeras is analogous to the overall delay in corneal reepithelialization after injury in irradiated mice versus non-irradiated mice, as reported by Ueno et al [5]; however, the delayed corneal healing was observed in both MRL and B6 mice. However, a limitation in the current study was the absence of syngeneic chimeras. While no differences have been previously described in syngeneic B6 or MRL chimeras in regards to wound healing [5], this could explain potential differences observed in the slight reduction in healing potential within the B6 chimeras in this study.

Previous studies have suggested that the improved healing of MRL mice is secondary to a muted inflammatory response [8]. Our results suggest that while a differential inflammatory response may play a role, it is not sufficient to enhance ear and/or cartilage repair since the donor MRL HSCs have not only reconstituted the systemic immune system (survival after lethal irradiation), but have also reconstituted the local joint environment. Thus, other cell types (progenitors or somatic cells) must make a significant contribution.

The results of the present and previous studies of MRL bone marrow chimeras and the transfer (or lack thereof) of repair capacity suggest that the “superhealer” phenotype may depend on additional cell types outside the bone marrow for enhanced tissue repair. Our results agree with this hypothesis, since MRL chimeras reconstituted with B6 bone marrow retained their super healer phenotype. Endogenous somatic or even terminally differentiated cells may play a role in MRL regenerative capacity. It has generally been found that the strain of the irradiated recipient, not the donor, determines the animal’s ability to heal ear punches, heart cryoinjuries or corneal wounds, and the authors concluded that the healing phenotype does not reside exclusively within bone marrow cells [4, 5].

In summary, bone marrow transplant from super-healer MRL mice into the non-healer B6 strain did not transfer the healing phenotype, either for ear punch wounds or for full thickness articular cartilage defects. The MRL regenerative phenotype may be dependent on cell types external to the bone marrow for enhanced tissue repair; however, additional transplantation studies on isolated and characterized cells are required to make any definitive conclusions.
